# Reversible regulation of ORC2 SUMOylation by PIAS4 and SENP2

**DOI:** 10.18632/oncotarget.19594

**Published:** 2017-07-26

**Authors:** Ronghua Wang, Fangming Liu, Yongxu Zhao, Dan Wu, Lihan Chen, Edward T.H. Yeh, Chao Huang

**Affiliations:** ^1^ The Central Lab at Shanghai Chest Hospital, Shanghai Jiao Tong University, Shanghai, China; ^2^ Institute of Health Sciences, Chinese Academy of Sciences-Jiaotong University School of Medicine, Shanghai, China; ^3^ Department of Internal Medicine, The University of Missouri, Columbia, MO, USA

**Keywords:** ORC2, SUMOylation, PIAS4, SENP2, polyploidy

## Abstract

The small ubiquitin-related modifier (SUMO) system is essential for smooth progression of cell cycle at the G2/M phase. Many centromeric proteins are reversibly SUMOylated to ensure proper chromosome segregation at the mitosis. SUMOylation of centromeric Origin Recognition Complex subunit 2 (ORC2) at the G2/M phase is essential in maintaining genome integrity. However, how ORC2 SUMOylation is regulated remains largely unclear. Here we show that ORC2 SUMOylation is reversibly controlled by SUMO E3 ligase PIAS4 and De-SUMOylase SENP2. Either depletion of PIAS4 or overexpression of SENP2 eliminated SUMOylation of ORC2 at the G/M phase and consequently resulted in abnormal centromeric histone H3 lysine 4 methylation. Cells stably expressing SENP2 protein or small interfering RNA for PIAS4 bypassed mitosis and endoreduplicated their genome to become polyploidy. Furthermore, percentage of polyploid cells is reduced after coexpression of ORC2-SUMO2 fusion protein. Thus, the proper regulation of ORC2 SUMOylation at the G2/M phase by PIAS4 and SENP2 is critical for smooth progression of the mitotic cycle of cells.

## INTRODUCTION

Accumulation of genetic and/or epigenetic alterations can cause cell transformation and initiate tumorigenesis. Genomic instability and its evolution in cancer cells increase tumor heterogeneity and result in an ever more aggressive phenotype [1, 2]. One of the mechanisms for evolution of genomic instability is a transient phase of polyploidization or tetraploidization, which may result from endoreduplication (DNA replication without mitosis), endomitosis (karyokinesis without cytokinesis) or aberrant cell fusion [3–6]. Tetraploid cells are prone to undergo asymmetric cell division, which can cause chromosome loss and aneuploidization [7, 8].

In normal cells, a variety of endogenous tumor suppressor genes prevent the generation of tetraploid cells. For instance, p53 is critical for initiates senescence or apoptosis induced by formation of tetraploidy that is caused by cell fusion, endoreplication, or endomitosis [9–11]. Therefore, inactivation of the p53 signaling pathway is a prerequisite for tetraploidization [12]. Moreover, activation of p38/MAP signaling pathway, pro-apoptotic gene Bax and cell-cycle regulated genes p21 and APC can result in the elimination of the tetraploid cell by programmed cell death [10, 12–16]. Symmetrically, overexpression of oncogene products such as Aurora A kinase or Polo-Like Kinase 1 (PLK1) induces tetraploidization [17, 18]. Tetraploidy and/or polyploidy promote the chromosomal rearrangements, translocations or gene amplifications that are major forces for cancer evolution and eventually results in aneuploidy and tumor heterogeneity, which is a hallmark of solid human cancer [5, 19].

SUMO proteins are small ubiquitin-like modifiers that covalently conjugated to cellular proteins [20]. Although SUMO E1 enzyme (SAE1/SAE2) and E2 enzyme Ubc9 are sufficient for *in vitro* SUMO modification of many proteins, SUMO E3 ligases play an crucial regulatory role *in vivo* by increasing the SUMOylation efficiency and also by determining the substrate specificity [21]. Overexpression or loss of SUMO E3 ligases function has fundamental impactions on almost every aspect of cell function [20, 22, 23]. A conservative group of SUMO E3 ligases has been found in all eukaryotes and contains a RING-finger like domain called SP-RING domain, which is responsible for recruiting Ubc9 [22, 24]. The SP-RING E3 ligases include the PIAS family proteins (PIAS1, PIASxα, PIASxβ, PIAS3, and PIAS4) in vertebrates and the Siz family proteins (Siz1 and Siz2) in *Saccharomyces cerevisiae* [24–26].

De-SUMOylation is essential to ensure the reversible nature of SUMO conjugation [27, 28]. SUMO isopeptidases (Ulps/SENPs) are responsible for both processing maturation of SUMO molecules and deconjugating the SUMOs from their substrates [27]. There are six different isopeptidases (SENP1, SENP2, SENP3, SENP5, SENP6, and SENP7) in human cells [20, 28]. SENP1 and SENP2 are most closely related to each other and catalyze both processing and deconjugation of SUMO-1 and SUMO-2/3 [29, 30]. In addition, both SENP1 and SENP2 are associated with the nuclear pore complex (NPC) and have a cellular distribution throughout the nucleus [31–33]. Dysregulation of SUMOylation and/or De-SUMOylation has been implicated in human diseases including various types of cancer [34].

Origin Recognition Complex (ORC) contains six conserved subunits ORC1–6 and is essential for the initiation of DNA replication in diverse organisms [35]. In addition to its role in establishing pre-RCs on chromosomes prior to DNA replication, ORC subunits are involved in other chromosome-associated processes [35, 36]. ORC2 localizes to centrosome and centromere for proper chromatin segregation at the G2/M phase [37]. ORC3 interacts with HP1 at heterochromatin foci to facilitate organizing higher chromatin structure [38]. ORC6 binds to the outer kinetochore during mitosis and localizes to the midplane of cell division in anaphase where it is required for cytokinesis via interaction with a septin protein [39]. Functions and localizations of ORC subunits are also regulated by posttranslational modifications such as phosphorylation and SUMOylation [40–42].

We have shown previously that ORC2 is SUMOylated at the G2/M phase of cell cycle and SUMOylation of ORC2 is critical for smooth transition of mitosis [42]; however, how ORC2 SUMOylation is controlled during cell cycle progression is unknown. Here, we show that ORC2 SUMOylation is reversibly regulated by SUMO E3 ligase PIAS4 and De-SUMOylase SENP2 at the G2/M phase of cell cycle. Loss of PIAS4 or overexpression of SENP2 in the cell results in formation of polyploidy, which can be partially rescued by ORC2-SUMO2 fusion protein. Our findings reveal that PIAS4 and SENP2 exert their cell cycle regulation functions partially through regulation of ORC2 SUMOylation.

## RESULTS

### PIAS4 and SENP2 control SUMOylation status of ORC2 at the mitosis

**Figure 1:**
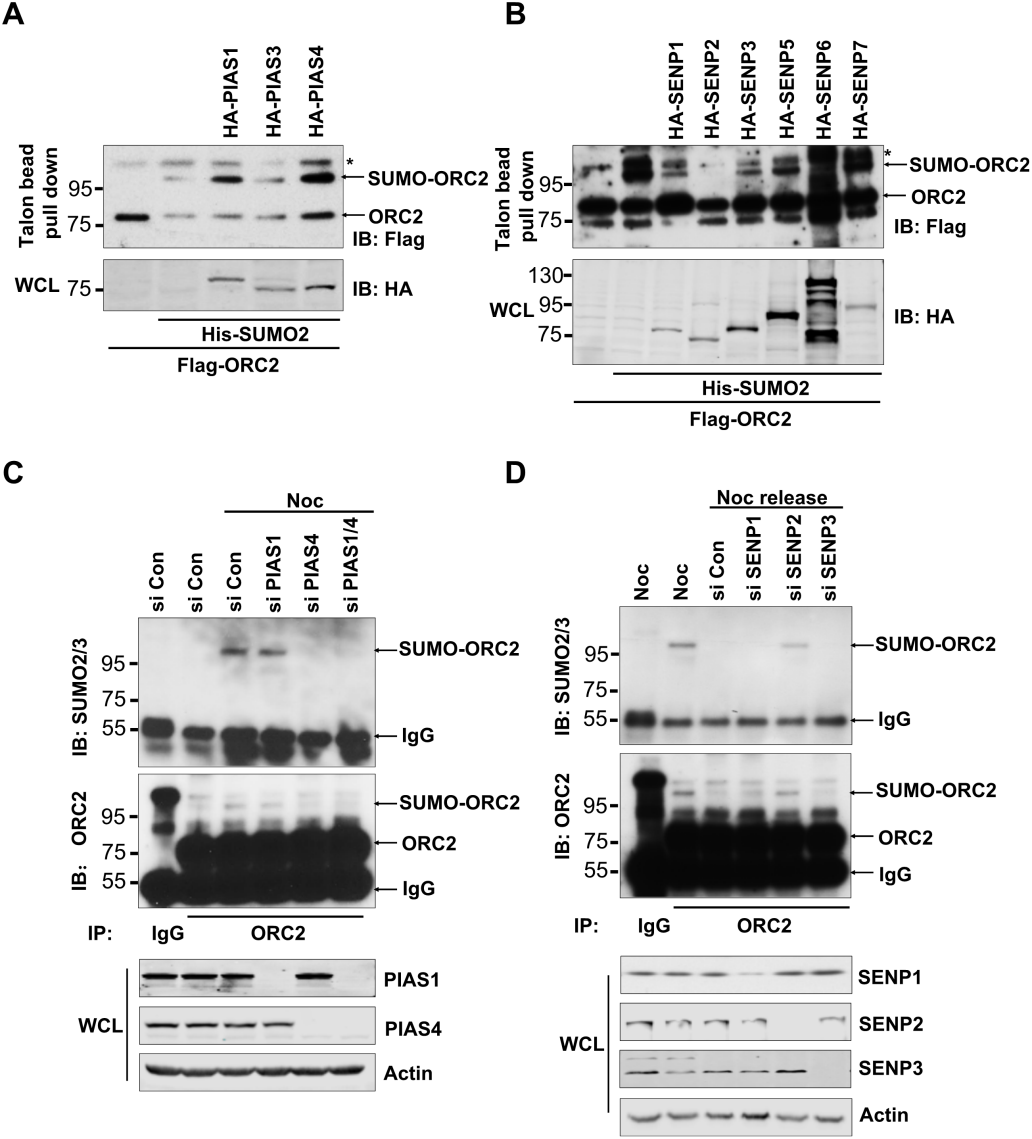
Cell-cycle regulated ORC2 SUMOylation by PIAS4 and SENP2 **(A)** U2OS cells were transfected with indicated plasmids. Talon beads pull-down was performed and precipitates were blotted with anti-FLAG antibody. Whole cell lysates (WCL) were blotted with HA antibody. **(B)** U2OS cells were transfected with indicated plasmids. Talon beads pull-down was performed and precipitates were blotted with anti-FLAG antibody. Whole cell lysates (WCL) were blotted with HA antibody. **(C)** U2OS cells were transfected with the indicated siRNA or control siRNA and treated with nocodazole for 24 hours. Cells were fractionated and the chromatin fraction was subjected to immunoprecipitation with ORC2 antibody. Immunoprecipitates were blotted with ORC2 or SUMO2 antibody. **(D)** U2OS cells were transfected with the indicated siRNA or control siRNA and treated with nocodazole for 24 hours, and then were released into fresh media for 2 hours. Cells were fractionated and the chromatin fraction was subjected to immunoprecipitation with ORC2 antibody. Immunoprecipitates were blotted with ORC2 or SUMO2 antibody. In all the figures, asterisks are indicated as non-specific bands. Arrows are indicated as SUMOylated ORC2, non-SUMOylated ORC2, or IgG.

Origin recognition complex subunit 2 (ORC2) is SUMOylated at the G2/M phase of the cell cycle [42]. To search for the SUMO E3 ligase and DeSUMOylase that are responsible for regulation of ORC2 SUMOylation, various SUMO E3 ligases or DeSUMOylases were overexpressed in U2OS cells (Figure 1A, 1B). Overexpression of SUMO E3 ligase PIAS 1 or PIAS 4, but not PIAS3, enhanced SUMOylation level of endogenous or overexpressed ORC2 (Figure 1A and Supplementary Figure 1A). By contrast, overexpression of DeSUMOylases SENP1, SENP2, or SENP3 reduced SUMOylation level of endogenous or overexpressed ORC2 (Figure 1B and Supplementary Figure 1B). SENP2 catalytic mutant lost de-SUMOylation activity on ORC2 (Supplementary Figure 1B). To further identify SUMO E3 ligase of ORC2, PIAS1 or PIAS4, or both, was knocked down in nocodazole-treated U2OS cells. ORC2 was immunoprecipitated and western blot with anti-ORC2 or anti-SUMO2/3 antibody showed that only depletion of PIAS4 reduced SUMOylated ORC2 at the G2/M phase (Figure 1C). We have previously shown that SUMOylation of ORC2 disappeared after exit of cell cycle from mitosis [42]. Therefore, DeSUMOylase SENP1, SENP2 or SENP3 was knocked down separately in U2OS cells released from nocodazole arrest. Immunoprecipitation with ORC2 antibody and western blot with anti-ORC2 or anti-SUMO2/3 antibody showed that depletion of SENP2, but not SENP1 or SENP3, enhanced SUMOylation level of ORC2 (Figure 1D). Altogether, these results clearly showed that ORC2 is SUMOylated by PIAS4 at the G2/M phase and is deSUMOylated by SENP2 upon exit of mitosis.

### PIAS4 and SENP2 sequentially interact with ORC2 throughout mitosis

**Figure 2:**
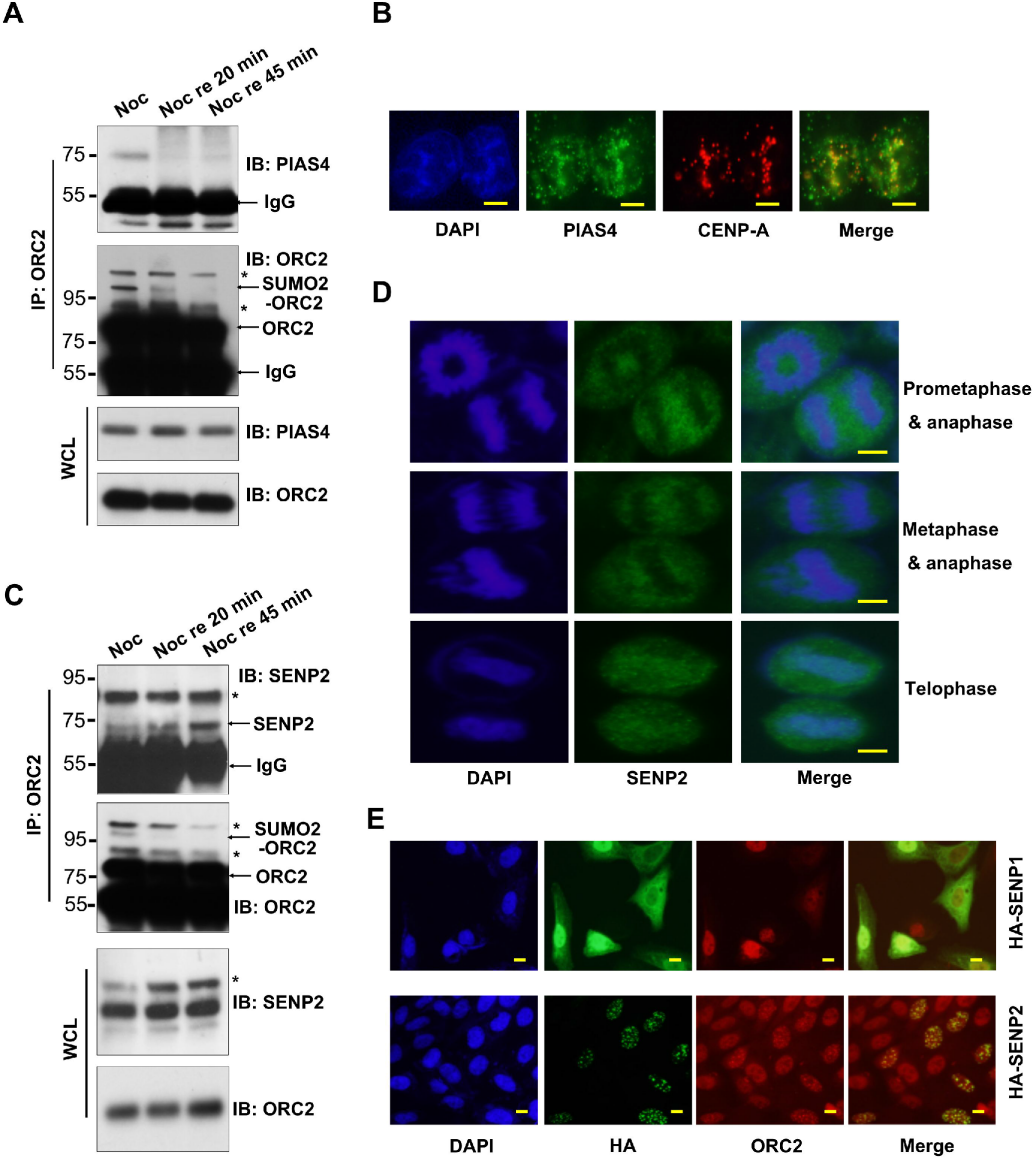
Dynamic interaction between ORC2 and PIAS4 or SENP2 **(A)** U2OS cells were treated with nocodazole for 24 hours and then released into fresh medium for indicated times. Immunoprecipitation was performed with ORC2 antibody and immunoprecipitates were blotted with ORC2 or PIAS4 antibody. Asterisks are indicated as non-specific bands and arrows are indicated as SUMOylated ORC2, non-SUMOylated ORC2, or IgG. **(B)** U2OS cells were treated with RO3306 and immunostaining was performed with PIAS4 antibody (green) and CENP-A antibody (red). Nuclei were stained with DAPI (blue). Scale bar: 10uM. **(C)** U2OS cells were treated with nocodazole for 24 hours and then released into fresh medium for indicated times as in (A). Immunoprecipitation was performed with ORC2 antibody and immunoprecipitates were blotted with ORC2 or SENP2 antibody. Asterisks are indicated as non-specific bands and arrows are indicated as SUMOylated ORC2, non-SUMOylated ORC2, or IgG. **(D)** U2OS cells were immunostained with SENP2 antibody. SENP2 staining (green) at different stages of mitosis was observed by microscope. Nuclei were stained with DAPI (blue). Scale bar: 10uM. **(E)** U2OS cells were transiently transfected with indicated plasmids. Cells were immunostained with HA antibody (green) and ORC2 antibody (red). Nuclei were stained with DAPI (blue). Scale bar: 10uM.

To further clarify regulation of ORC2 SUMOylation, U2OS cells were treated with nocodazole and then released into fresh medium for different time. Immunoprecipitation with ORC2 antibody western blot with anti-PIAS4 antibody showed that PIAS4 bound to ORC2 in nocodazole-treated cells (Figure 2A). However, PIAS4 dissociated from ORC2 upon cells were released into fresh medium, which may result in gradually loss of ORC2 SUMOylation (Figure 2A). To further confirm interaction between PIAS4 and ORC2, cells were arrested at the G2 phase by CDK1 inhibitor RO3306. Immunostaining showed that at the G2 phase, PIAS4 colocalized with CENP-A at centromeric chromatin (Figure 2B), where multiple proteins including ORC2 are SUMOylated [37, 43]. PIAS4 dissociated from centromeric region as cells entered the G1 phase (Supplementary Figure 1C). On the contrary, SENP2 gradually bound to ORC2 once cells were released from nocodazole arrest (Figure 2C). Simultaneously, SUMOylation level of ORC2 gradually decreased upon release of cells into fresh medium (Figure 2A, 2C). Immunostaining showed that SENP2 did not locate to chromosome at the prometaphase, metaphase and anaphase of cell cycle (Figure 2D). However, SENP2 relocated to chromosome at the telophase (Figure 2D), consistent with the western blot results of interaction between SENP2 and ORC2 (Figure 2C). Interestingly, overexpression of SENP2 induced formation of ORC2 foci at the interphase, which colocalized with SENP2 (Figure 2E) [44]. Altogether, all these data indicate that mitotic SUMOylation of ORC2 is dynamically regulated by sequential binding of ORC2 with PIAS4 and SENP2 during the course of mitosis.

### Depletion of PIAS4 or overexpression of SENP2 causes abnormal mitotic cycle

**Figure 3:**
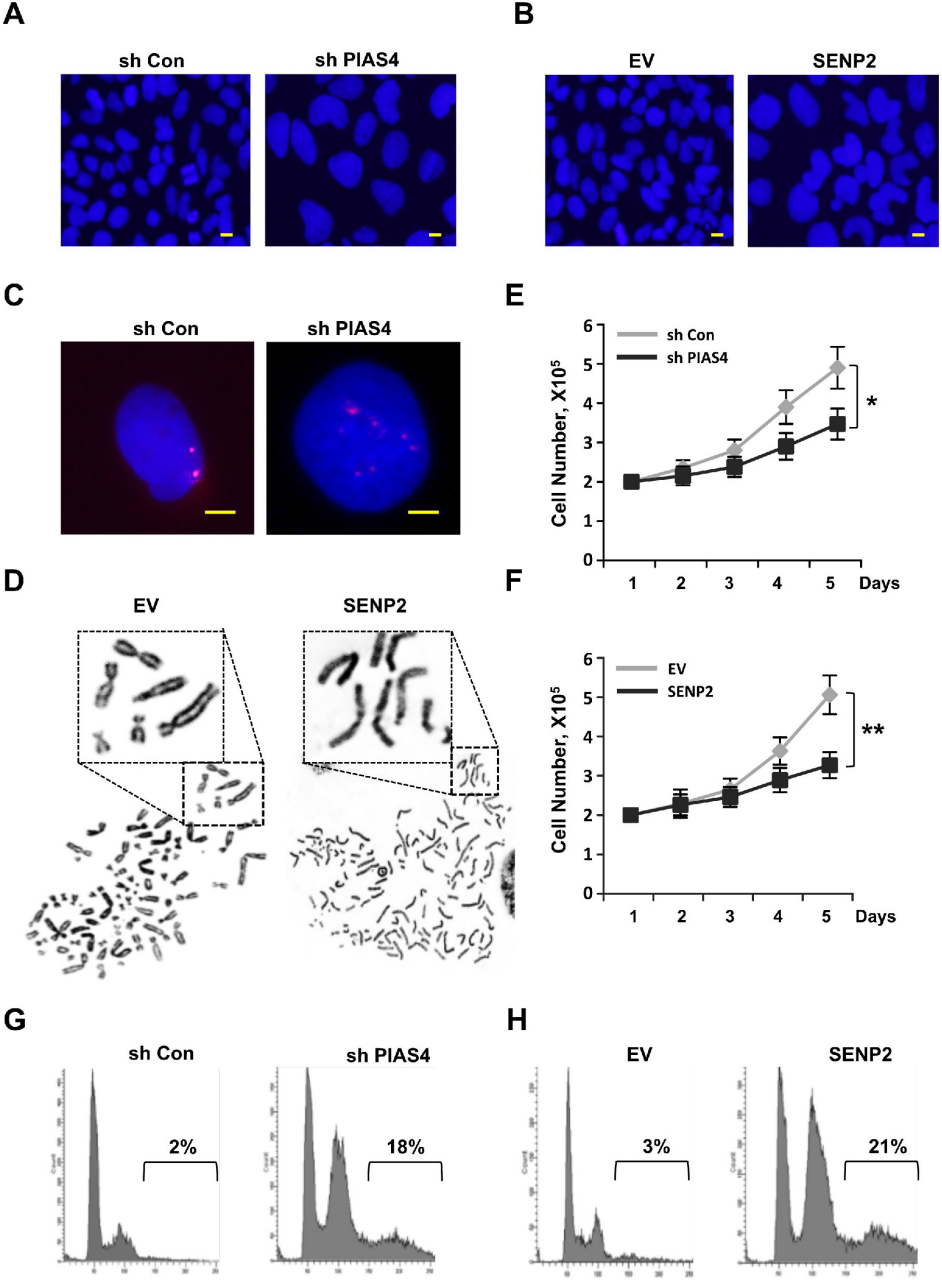
Deregulated expression of PIAS4 or SENP2 results in abnormal mitotic cycle **(A** and **B)** U2OS cells stably expressing PIAS4 shRNA (A) or SENP2 (B) were stained with DAPI. Nuclear morphology was shown. Scale bar: 10uM. **(C)** Duplication of chromosome. DNA FISH using a chromosome 19 satellite repeat probe (red) in cells stably expressing PIAS4 shRNA. Scale bar: 10uM. **(D)** Typical image of metaphase chromosome spread of cells stably expressing SENP2. **(E** and **F)** 2X10^5^ PIAS4-knocking down cells or SENP2-overexpressing cells or control cells were plated and cell number was counted every day for five days. The graph shows the average of three independent experiments; mean ± S.D.; *, p < 0.05; **, p < 0.01. **(G** and **H)** Cell cycle profiles of PIAS4 shRNA-expressing cells (G) and SENP2-overexpressing cells (H) are shown.

Loss of ORC2 SUMOylation can result in temporally G2/M phase arrest and formation of polyploidy [42]. To test whether PIAS4 or SENP2 misregulation has the similar impacts on cell cycle progression, we constructed two cell lines that either stably expresses short hairpin RNA (shRNA) for PIAS4 or stably overexpresses SENP2. Depletion of PIAS4 or overexpression of SENP2 produced abnormal nuclear morphology in cells with big, dumbbell-shaped nuclei, especially in SENP2-overexpressing cells (Figure 3A, 3B). FISH analysis of PIAS4-depleted cells or metaphase chromosome spread of SENP2-overexpressing cells showed that chromosomes in both cells were overduplicated (Figure 3C, 3D). Interestingly, chromosomes remain unseparated in SENP2-overexpressing cells, which were different from that in ORC2 SUMOylation mutant cells [42]. Depletion of PIAS4 or overexpression of SENP2 reduced cell proliferation compared with control cells (Figure 3E, 3F). To quantify the cells with DNA content >4C in both cell lines, Fluorescence activated cell sorting (FACS) was performed. Results showed that both cell lines were arrested at the G2/M phase and there were 18% and 21% cells with DNA content >4C in PIAS4-depleted cells or SENP2-overexpressing cells, respectively (Figure 3G, 3H). All these results indicated that cells were arrested at the G2/M phase and a significant portion of polyploidy was formed in cells with stable depletion of PIAS4 or overexpression of SENP2.

### Formation of polyploidy in cell population was caused by bypass of mitosis

**Figure 4:**
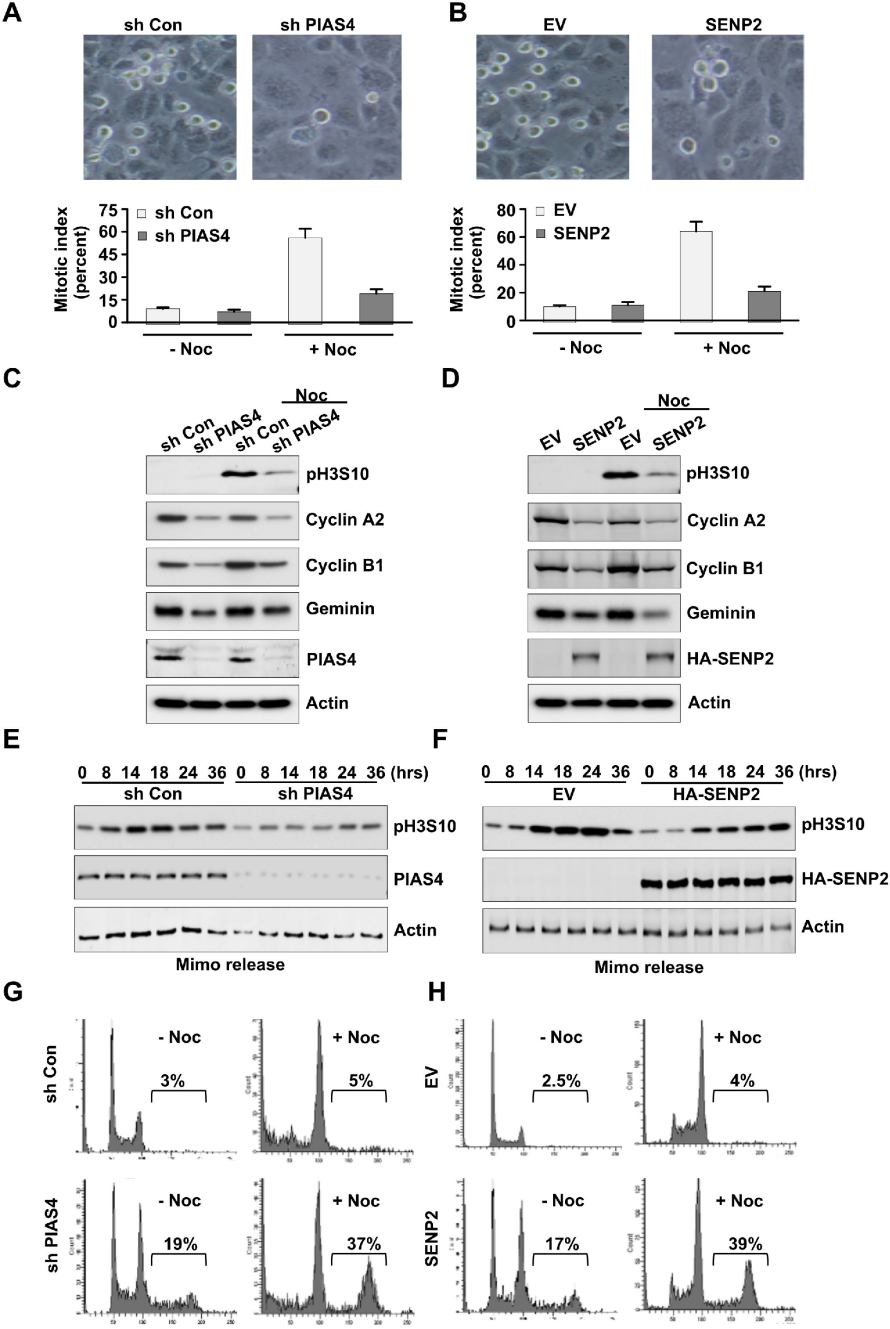
Deregulated expression of PIAS4 or SENP2 results in by pass of mitosis **(A** and **B)** U2OS cells stably expressing PIAS4 shRNA (A) or SENP2 (B) were treated with or without nocodazole for 20 hours. Cell image (*upper panels*) and Mitotic index (*lower panels*) was shown. **(C** and **D)** U2OS cells stably expressing PIAS4 shRNA (C) or SENP2 (D) were treated with or without nocodazole for 20 hours. Western blot was performed to detect expressions of different proteins as indicated. **(E** and **F)** U2OS cells stably expressing PIAS4 shRNA (E) or SENP2 (F) were treated with mimosine for 24 hours. Cells were released into fresh medium for different times. Western blot was performed to detect expression of phosphorylated histone H3 Ser 10. **(G** and **H)** U2OS cells stably expressing PIAS4 shRNA (G) or SENP2 (H) were treated with or without nocodazole for 24 hours. Cell cycle profiles were analyzed by FACS.

To investigate the mechanisms of polyploidy formation, PIAS4-depleted cells or SENP2-overexpressing cells were treated with nocodazole for 20 hours, and then mitotic index was calculated. Results showed that mitotic index in control cells was greatly increased under the nocodazole treatment condition; however, mitotic index was only slightly increased in PIAS4-depleted cells or SENP2-overexpressing cells (Figure 4A, 4B). Western blot showed that expression level of mitosis marker phosphorylated histone H3 Ser 10 in control shRNA transfected cells or empty vector transfected cells was greatly increased with nocodazole treatment. However, level of phosphorylated H3 Ser 10 was only slightly increased in PIAS4-depleted cells or SENP2-overexpressing cells by nocodazole (Figure 4C, 4D). Degradation of the G2/M phase markers Cyclin A or Cyclin B is required for bypass of mitosis [45, 46]. We checked the expressions of Cyclin A and Cyclin B and the results showed their reduced levels after depletion of PIAS4 or overexpression of SENP2 (Figure 4C, 4D). Furthermore, expression of Geminin, a DNA replication inhibitor, was decreased in PIAS4-depleted cells or SENP2-overexpressing cells (Figure 4C, 4D), suggesting overduplication of genome in the cells [47]. Next, cells were arrested at late G1 phase by mimosine and then released into fresh medium for different times. Phosphorylation of H3 Ser 10 in control cells reached its maximum level between 14-24 hours after release. However, H3 Ser 10 phosphorylation level in PIAS4-depleted cells or SENP2-overexpressing cells only slightly increased after released for even 24-36 hours (Figure 4E, 4F). FACS analysis revealed that percentage of polyploidy in PIAS4-depleted cells or SENP2-overexpressing cells was greatly increased by nocodazole treatment for 24 hours (Figure 4G, 4H). Collectively, these data indicated that depletion of PIAS4 or overexpression of SENP2 caused temporally G2/M phase arrest; however, longer arrest resulted in bypass of mitosis, which eventually led to overduplication of genome and formation of polyploidy.

### Abnormal centromeric histone H3K4 methylation in cells with misregulated expression of PIAS4 or SENP2

**Figure 5:**
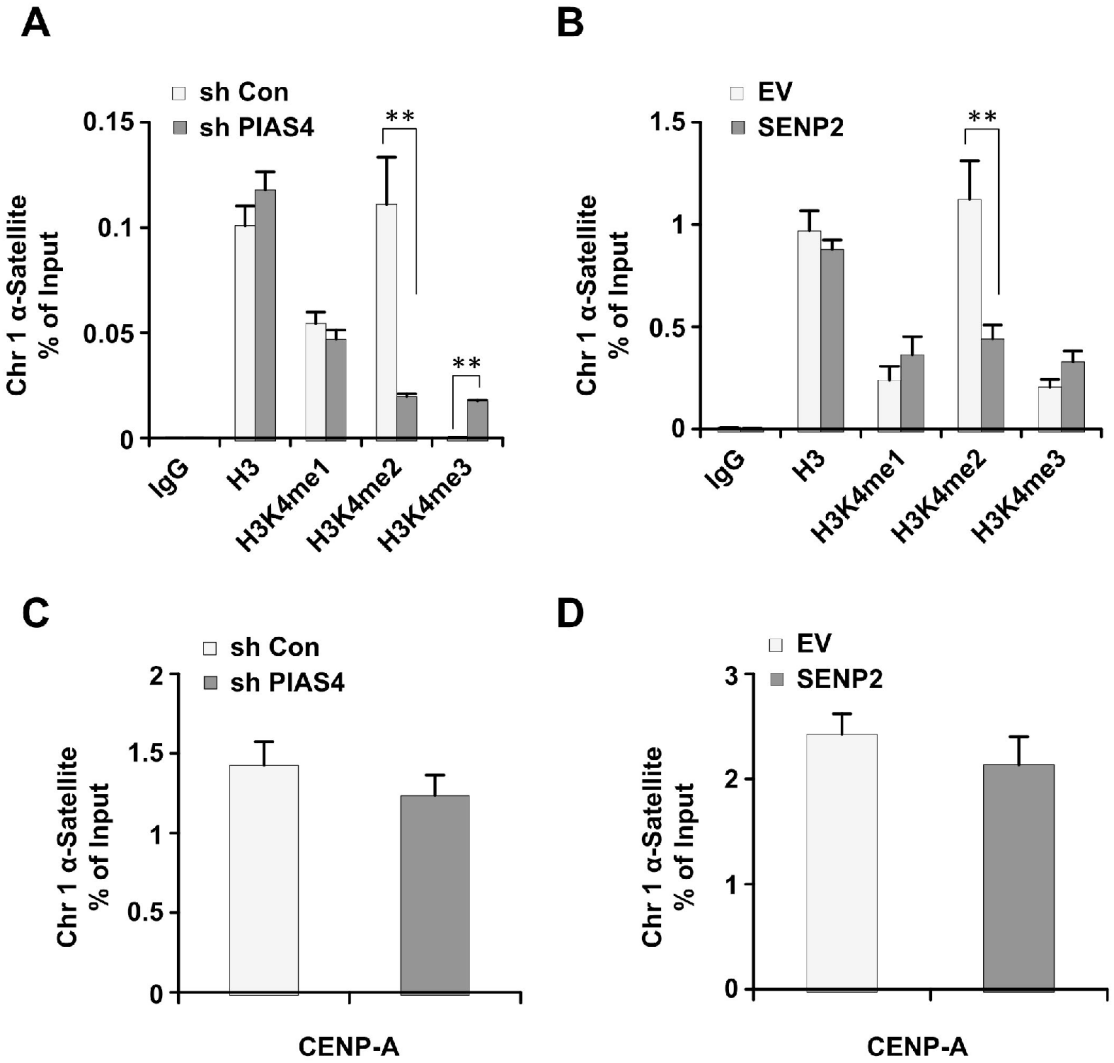
Depletion of PIAS4 or overexpression of SENP2 leads to abnormal histone H3K4 methylation **(A** and **B)** The relative occupancy of H3, H3K4me1, H3K4me2, and H3K4me3 within α-satellite of chromosome 1 was analyzed using ChIP-PCR in cells stably expressing PIAS4 shRNA (A) or SENP2 (B). The graph shows the average of three independent experiments; mean ± S.D. **, p < 0.01. **(C** and **D)** The relative occupancy of CENP-A within α-satellite of chromosome 1 was analyzed using ChIP-PCR in cells stably expressing PIAS4 shRNA (C) or SENP2 (D). The data represent the means and SEM of three independent experiments.

Centromeric histone H3K4 methylation is misregulated in ORC2 SUMOylation mutant cells [42]. Therefore, we would like to see whether long term depletion of PIAS4 or overexpression of SENP2 has similar effects on centromeric histone H3K4 methylation. Results from chromatin immunoprecipitation analysis showed that level of centromeric H3K4me2 is reduced, whereas centromeric H3K4me3 is increased, in PIAS4-depleted or SENP2-overexpressing cells (Figure 5A, 5B). Level of centromeric H3K4me1 did not show significant change in both cell lines compared to control cells (Figure 5A, 5B). Abundance of centromeric marker protein CENP-A did not show significant difference between control cells and PIAS4-depleted or SENP2-overexpressing cells (Figure 5C, 5D). Altogether, these results showed that centromeric histone H3K4 methylation was dysregulated by depletion of PIAS4 or overexpression of SENP2, reminiscent of the centromeric histone modifications in ORC2 SUMOylation mutant cells [42].

### Abnormal mitotic cycle of PIAS4-depleted or SENP2-overexpressing cells were partially rescued by overexpression of ORC2-SUMO2 fusion protein

**Figure 6:**
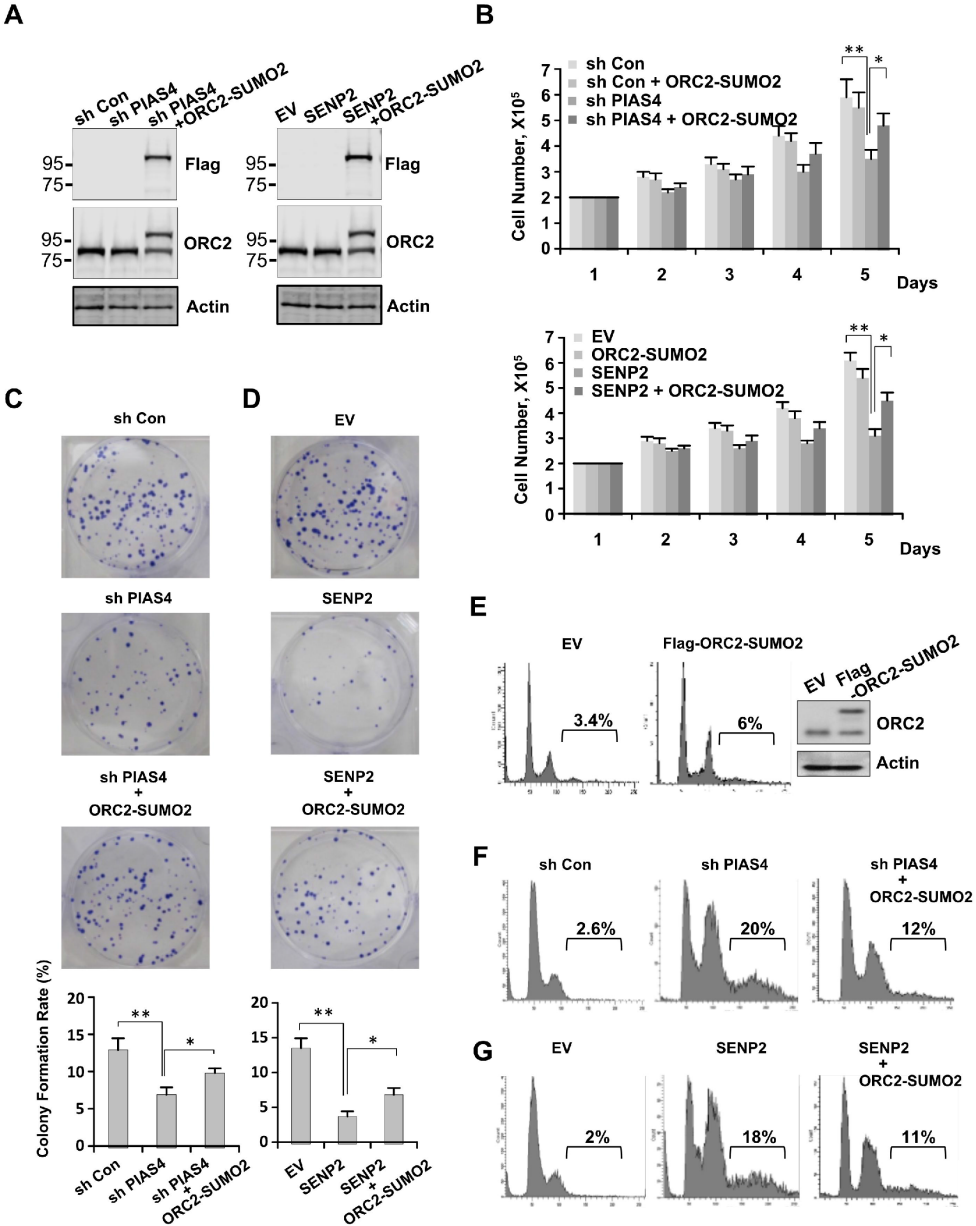
Overexpression of ORC2-SUMO2 fusion protein partially reversed phenotype of cells stably expressing PIAS4 shRNA or SENP2 **(A)** Various U2OS cells lines as indicated were subjected to western blot analysis with indicated antibodies. **(B)** 2X10^5^ U2OS cells stably expressing control shRNA, control shRNA/ORC2-SUMO2, PIAS4 shRNA or PIAS4 shRNA/ORC2-SUMO2 as indicated (*upper panel*) or EV, EV/ORC2-SUMO2, SENP2 or SENP2/ORC2-SUMO2 as indicated (*lower panel*) were plated and cell number was counted every day for five days. The graph shows the average of three independent experiments; mean ± SD. *, p < 0.05; **, p < 0.01. **(C** and **D)** 1000 U2OS cells stably expressing control shRNA, PIAS4 shRNA or PIAS4 shRNA/ORC2-SUMO2 (C) or EV, SENP2 or SENP2/ORC2-SUMO2 (D) were seeded per well of a six-well plate. After incubation for 10–14 days, cells were stained with crystal violet (*upper panel*). Analysis of colony formation rates of cells (*lower panel*). Data are means ± SD of three independent experiments. *, p < 0.05; **, p < 0.01. **(E)** U2OS cells were transient transfected with Flag tagged ORC2-SUMO2. Western blot and FACS were performed to detect expression of Flag-ORC2-SUMO2 protein and cell cycle profile, respectively. **(F** and **G)** Cell cycle profiles of cells stably expressing control shRNA, PIAS4 shRNA or PIAS4 shRNA/ORC2-SUMO2 (F) or EV, SENP2 or SENP2/ORC2-SUMO2 (G) were analyzed by FACS.

To test whether loss of ORC2 SUMOylation play a role in cell cycle defects caused by abnormal expression of PIAS4 or SENP2, both PIAS4-depleted cell line and SENP2-overexpressing cell line were transfected with expression vector for fusion protein ORC2-SUMO2. Expression level of ORC2-SUMO2 in both cell lines was similar with that of endogenous ORC2 (Figure 6A). Cell proliferation and cell viability assay showed that impaired cell growth caused by depletion of PIAS4 or overexpression of SENP2 was partially reversed by overexpression of ORC2-SUMO2 protein (Figure 6B and Supplementary Figure 2A). Colony formation assay showed that depletion of PIAS4 or overexpression of SENP2 reduced colony formation rates of cells, whereas overexpression of ORC2-SUMO2 partially rescued the ability of both cell lines to form colonies (Figure 6C, 6D). Next, FACS analysis was performed to further investigate functional effects of ORC2-SUMO2 on cell cycle. Overexpression of ORC2-SUMO2 did not significantly affect cell cycle profile in U2OS cells (Figure 6E). However, percentage of polyploidy in PIAS4-depleted or SENP2-overexpressing cells was significantly reduced from 20% to 12% and 18% to 11%, respectively, by overexpression of ORC2-SUMO2 fusion protein in both cell lines (Figure 6F, 6G). Interestingly, knocking down of SENP2 did not affect mitotic cell cycle as previously reported [44], suggesting de-SUMOylation of ORC2 by SENP2 is not required for cells to exit mitosis (Supplementary Figure 2B). Altogether, these results suggest that abnormal mitotic cycle in PIAS4-depleted or SENP2-overexpressing cell line was at least partially caused by loss of ORC2 SUMOylation.

## DISCUSSION

In this study we showed that SUMOylation of ORC2 was reversibly regulated by SUMO E3 ligase PIAS4 and De-SUMOylase SENP2 at the G2/M phase. Either downregulation of PIAS4 or overexpression of SENP2 resulted in accumulation of polyploidy in cell population. Formation of polyploidy is partially caused by loss of ORC2 SUMOylation, as overexpression of ORC2-SUMO2 fusion protein in PIAS4-depleted cells or in SENP2-overexpressing cells partially reduced percentage of polyploid cells. Our results revealed that the function of PIAS4 and SENP2 in controlling cell cycle progression and genomic stability is at least mediated by direct regulation of ORC2 SUMOylation.

ORC2, along with other ORC subunits, are first discovered for DNA replication initiation by binding to replication origins to form pre-RC complex at late G1 and early S phase in mammalian cells [35]. However, ORC2 is not required for DNA replication initiation once pre-RC is formed, and gradually dissociates from DNA replication origins and localizes to centromere at the G2/M phase [37, 40, 48]. ORC2 also localizes at telomeric region and is essential for telomere homeostasis [49, 50]. Depletion of ORC2 has been shown to result in mitotic arrest due to defects in chromosome condensation [37]. In yeast, ORC2 is required for sister-chromatid cohesion. Downregulation of ORC2 causes cell cycle to be arrested at the G2/M phase [51]. The Drosophila ORC2 mutants k43 also causes G2/M phase arrest with abnormally condensed chromosomes [52]. These studies indicate a critical role of ORC2 in regulation of mitotic cycle and genome integrity at the G2/M phase.

Posttranslational modifications, such as phosphorylation, ubiquitination and SUMOylation, have been demonstrated to be essential for chromosomal integrity during mitosis [22, 53, 54]. Like phosphorylation, evolutionally conserved protein SUMOylation at mitotic centromeres and kinetochores are consistent with a model that SUMOylation functions as a master regulator of centromere and kinetochore activities during mitosis in both invertebrate and vertebrate cells [43, 55, 56]. ORC2 has been shown to be subjected to phosphorylation and SUMOylation [40, 42]. Defects in ORC2 SUMOylation result in dysfunction of chromosome segregation and eventually lead to formation of polyploidy [42]. However, how SUMOyaltion of ORC2 is regulated at the G2/M phase is unclear.

Centromeric PIASy is critical for the accumulation of SUMO2/3 conjugates at the inner centromere region of mitotic chromosomes, and is also required for SUMO2/3 modification of Topoisomerase IIα and its subsequent localization to centromeres in Xenopus extracts and human cells [43, 57–59]. However, another SUMO E3 ligase RanBP2 has also been found to be essential for SUMOylation of Topoisomerase IIα in mice [60]. PIASy-mediated SUMO2/3 conjugation of Topoisomerase IIα significantly inhibits the decatenation activity of Topoisomerase IIα in Xenopus egg extracts [61]. After the sister chromosomes are accurately aligned at the early anaphase, PIAS4 dissociates from the kinetochores. Subsequently, due to the absence of PIAS4 and the presence of multiple SENPs, Topoisomerase IIα is not conjugated by SUMO2/3 any more during anaphase, allowing enhanced decatenation activity and correct chromosome segregation [34, 59]. Other than Topoisomerase IIα, multiple centromeric proteins, such as BubR1, Borealin, CENP-E, ORC2, etc., are conjugated by SUMO1/2/3, suggesting mitotic abnormality caused by down regulation of PIAS4 or RanBp2 cannot be attributed to a single SUMO substrate [34, 42]. Consistently, depletion of SUMO E2 Ubc9 also results in a loss of global protein SUMOylation and abnormal cell cycle progression at the G2/M phase [56]. We showed here that long term depletion of PIAS4 resulted in by pass of mitosis and formation of polyploidy in U2OS cells. This phenotype can be partially rescued by overexpression of ORC2-SUMO2 fusion protein, suggesting PIAS4-regulated ORC2 SUMOylation play a role in maintaining genome stability.

SENP1, SENP2 and SENP3 have been found to exert spatial and temporal regulation of protein SUMOylation in mitosis [44, 56, 62]. We showed here that SUMOylation of ORC2 was affected by depletion of SENP2, not by SENP1 or SENP3, suggesting differential substrates specificity for various SENPs at mitotic centromere. SENP2 has been shown to be different from SENP1 and SENP3 in its ability to cause a mitotic, prometaphase arrest when overexpressed in cultured mammalian cells [56]. The ability of overexpressed SENP2 to cause cell cycle arrest is due to a unique association with kinetochores during mitosis that depends on interactions with the Nup107-160 subcomplex of the nuclear pore complex (NPC) and/or defects in targeting CENP-E to kinetochores in mammalian cells [44, 56]. SENP2 overexpression causes the disappearance of SUMO2/3 signals on mitotic centromeres and kinetochores without affecting the SUMO1-RanGAP1 staining on mitotic spindles [56]. However, SENP2 overexpression does not affect the overall structure and function of kinetochores, as shown by correct localization of centromere and kinetochore proteins (including Aurora B, CENP-B, CENP-C, CENP-F and ORC2) on mitotic chromosomes in SENP2 overexpressing cells [42, 56, 63]. We showed here that stable overexpression of SENP2 in the cells results in bypass of mitosis and formation of polyploidy, which is partially caused by loss of ORC2 SUMOylation. It is interestingly to note that in our study overexpression of SENP2 caused formation of nuclear foci that contained ORC2 protein at the interphase. SUMOylation has been show to exert negative effects on activation of DNA replication origins [64]. Therefore, whether De-SUMOylation of ORC2 by SENP2 at the G1/S phase contributes to DNA replication initiation or elongation deserves further investigation.

In summary, we showed here that PIAS4 bind to ORC2 at the M phase to mediate ORC2 SUMOylation and SENP2 interacted with SUMOylated ORC2 for De-SUMOylation upon cells exit M phase. The data presented here suggest the balance between dynamic ORC2 SUMOylation and De-SUMOylation maintained by SUMO E3 ligase and De-SUMOylase is essential for normal progression of cell cycle and genome stability.

## MATERIALS AND METHODS

### Cell culture and generation of stable expressing cells

U2OS cells purchased from ATCC were grown under standard tissue-culture conditions. To generate U2OS cells continuously expressing PIAS4 shRNA, PIAS shRNA (sc-40852-SH) was purchased from Santa Cruz Biotechnology and selected with puromycin (0.5 mg/ml) after transfection of U2OS cells. For cells continuously expressing HA-SENP2 or Flag-ORC2-SUMO2, PQCXIP vectors containing HA-SENP2 or Flag-ORC2-SUMO2 fragment were first transfected into Platinum A package cells (Cell Biolabs) using Fugene 6 transfection reagent (Roche). At 2 days after transfection, the supernatant was collected and filtered through a 0.45 mm filter. Medium (1 ml) was removed from a 3-cm plate with the U2OS cells at around 40%–50% confluence and 1ml of filtered supernatant was added with polybrene (final polybrene concentration, 8 mg/ml). U2OS cells were selected by puromycin (0.5 mg/ml) after 24 hr infection. Cells stable expressing PIAS shRNA or SENP2 were transfected with Flag-ORC2-SUMO2 plasmids, after 24 hours transfection cells were cultured under selection of both puromycin and G418 for over two weeks to make cells stable expressing PIAS4 shRNA and Flag-ORC2-SUMO2, or cells stable expressing SENP2 and Flag-ORC2-SUMO2.

### Cell fractionation, immunoprecipitation, and talon beads pull-down

Cell fractionation was performed as described previously [65]. Chromatin of cells was sonicated and digested with 0.2 U micrococcal nuclease (Sigma). Supernatant was used for routine immunoprecipitation and western blotting with anti-ORC2 (cat. #4736, Cell Signaling), anti-FLAG, anti-HA (Sigma), anti-SENP1 antibody (cat. #ab108981, Abcam), anti-SENP2 antibody (cat. #ab58418, Abcam), anti-SENP3 antibody (cat. #5591, Cell Signaling), anti-PIAS1 (cat. #ab32219, Abcam), anti-PIAS4 (cat. #ab211625, Abcam), anti-SUMO2/3 antibody (cat. #ab81371, Abcam), antibodies. Talon beads pull-down was performed as described previously [66].

### Fluorescence activated cell sorting (FACS)

FACS analysis for DNA content was performed using standard methods and PI DNA staining. Flow cytometry was performed using FACS caliber (Becton Dickinson), and FlowJo software was used for estimation of the percentage of cells in various phases of the cell cycle.

### Immunofluorescence

For HA-SENP1, HA-SENP2 staining, cultured U2OS cells were transient transfected with above HA tagged plasmids. Cells were stained with anti-HA antibody (cat. #H6908, Sigma). For endogenous SENP2 staining, cells were stained with anti-SENP2 antibody (cat. # ab58418, Abcam). For endogenous PIAS4 and CENP-A staining, U2OS cells were stained with anti-PIAS4 (cat. # ab211625, Abcam) and anti-CENP-A (cat. #2186, Cell Signaling). After incubation with the primary antibodies at room temperature for 1 hr, Alexa 488- and Alexa 594-labeled secondary antibodies (Life Technology) were added for 1 hr. Cells were then adhered to a slide with DAPI-staining Mounting Medium (Vector Labs). All samples were visualized with an Olympus fluorescence microscope and images were derived with the accompanying DP-BSW application software program.

### DNA FISH

DNA FISH were performed as described [42]. U2OS cells stably expressing PIAS4 shRNA were pre-extracted in cytoskeletal buffer (CSK: 100 mM NaCl, 300 mM sucrose, 3 mM MgCl 2, and 10 mM PIPES at pH 6.8) containing 0.5% Triton X-100 for 5 min on ice and fixed with 3.7% freshly prepared formaldehyde for 15 min at room temperature. The cells were washed in 1X PBS (pH 7.2) and heat denatured in 70% formamide and 2X saline sodium citrate (SSC) at 72°C for 5 min followed by hybridization with labeled chromosome 19-specific satellite probe (Agilent) in 2X SSC, 50% formamide, 10% dextran sulfate, yeast tRNA, and Cot-1 DNA overnight at 37°C.

### ChIP assay

Chromatin-IP was performed using the ChIP Assay Kit (Millipore) according to the manufacturer’s protocol by using anti-H3 (cat. #14269), anti-CENP-A (cat. #2186), anti-H3K4me1 (cat. #5326), anti-H3K4me2 (cat. #9725), and anti-H3K4me3 (cat. #8580). All antibodies are from Cell Signaling. Real-time PCR was performed to detect relative occupancy with the primers for chromosome 1 a-satellite DNA purchased from Cell Signaling.

### Metaphase chromosome spread

U2OS cells stably expressing HA-SENP2 were used for the assay. The experiments were performed according to previously reported [58]. In brief, cells were harvested by mitotic shake off and hypotonically swollen in 40% medium for 5 min at room temperature. Cells were fixed with freshly made Carnoy's solution (75% methanol, 25% acetic acid), and the fixative was changed several times. For spreading, cells in Carnoy's solution were dropped onto glass slides and dried at 37°C. Slides were stained with 5% Giemsa (Merck) at pH 6.8 for 7 min, washed briefly in ddH_2_O, air dried, and mounted with Entellan (Merck).

### Colony formation assay

Colony formation assay was performed as described previously [67]. In brief, cells at the exponential growth phase were harvested with trypsin-EDTA and counted using a hemocytometer. Following this, cells were diluted and seeded at about 1000 cells per well of a six-well plate, and then continuously incubated in new fresh medium at 37 °C in 5% humidified CO2. After incubation for 10–14 days, cells were washed with PBS twice, fixed with methanol for 15 min, and stained with 0.5% crystal violet for 15 min at room temperature. Visible colonies were counted. Colony formation rate = (number of colonies/number of seeded cells) × 100%.

### MTT assay

Cell viability was analyzed using the 3-(4, 5-dimethylthiazol-2-yl)-2, 5-diphenyltetrazolium bromide (MTT, Sango, China) assay, as previously described [68].

### Statistical analysis

SPSS 17.0 software was used for statistical analysis. Analysis of variance was applied for single- or multifactor variance analysis. A Student-Newman-Keuls test was used for the comparison of mean. A value of p < 0.05 was considered statistically significant.

## SUPPLEMENTARY MATERIALS FIGURES



## References

[1] Merlo LM, Pepper JW, Reid BJ, Maley CC (2006). Cancer as an evolutionary and ecological process. Nat Rev Cancer.

[2] Burrell RA, McGranahan N, Bartek J, Swanton C (2013). The causes and consequences of genetic heterogeneity in cancer evolution. Nature.

[3] Ogle BM, Cascalho M, Platt JL (2005). Biological implications of cell fusion. Nat Rev Mol Cell Biol.

[4] Shi Q, King RW (2005). Chromosome nondisjunction yields tetraploid rather than aneuploid cells in human cell lines. Nature.

[5] Davoli T, de Lange T (2011). The causes and consequences of polyploidy in normal development and cancer. Ann Rev Cell Dev Biol.

[6] Lee HO, Davidson JM, Duronio RJ (2009). Endoreplication: polyploidy with purpose. Genes Dev.

[7] Margolis RL (2005). Tetraploidy and tumor development. Cancer cell.

[8] Lacroix B, Maddox AS (2012). Cytokinesis, ploidy and aneuploidy. J Pathol.

[9] Sugimoto M, Gromley A, Sherr CJ (2006). Hzf, a p53-responsive gene, regulates maintenance of the G2 phase checkpoint induced by DNA damage. Mol Cell Biol.

[10] Aylon Y, Michael D, Shmueli A, Yabuta N, Nojima H, Oren M (2006). A positive feedback loop between the p53 and Lats2 tumor suppressors prevents tetraploidization. Genes Dev.

[11] Vitale I, Galluzzi L, Vivet S, Nanty L, Dessen P, Senovilla L, Olaussen KA, Lazar V, Prudhomme M, Golsteyn RM, Castedo M, Kroemer G (2007). Inhibition of Chk1 kills tetraploid tumor cells through a p53-dependent pathway. PLoS One.

[12] Ganem NJ, Pellman D (2007). Limiting the proliferation of polyploid cells. Cell.

[13] Castedo M, Coquelle A, Vivet S, Vitale I, Kauffmann A, Dessen P, Pequignot MO, Casares N, Valent A, Mouhamad S, Schmitt E, Modjtahedi N, Vainchenker W (2006). Apoptosis regulation in tetraploid cancer cells. EMBO J.

[14] Waldman T, Lengauer C, Kinzler KW, Vogelstein B (1996). Uncoupling of S phase and mitosis induced by anticancer agents in cells lacking p21. Nature.

[15] Tighe A, Johnson VL, Taylor SS (2004). Truncating APC mutations have dominant effects on proliferation, spindle checkpoint control, survival and chromosome stability. J Cell Sci.

[16] Zarubin T, Han J (2005). Activation and signaling of the p38 MAP kinase pathway. Cell Res.

[17] Wang X, Zhou YX, Qiao W, Tominaga Y, Ouchi M, Ouchi T, Deng CX (2006). Overexpression of aurora kinase A in mouse mammary epithelium induces genetic instability preceding mammary tumor formation. Oncogene.

[18] Incassati A, Patel D, McCance DJ (2006). Induction of tetraploidy through loss of p53 and upregulation of Plk1 by human papillomavirus type-16 E6. Oncogene.

[19] Kops GJ, Weaver BA, Cleveland DW (2005). On the road to cancer: aneuploidy and the mitotic checkpoint. Nat Rev Cancer.

[20] Yeh ET (2009). SUMOylation and De-SUMOylation: wrestling with life's processes. J Biol Chem.

[21] Flotho A, Melchior F (2013). Sumoylation: a regulatory protein modification in health and disease. Annu Rev Biochem.

[22] Dasso M (2008). Emerging roles of the SUMO pathway in mitosis. Cell Div.

[23] Hay RT (2005). SUMO: a history of modification. Mol Cell.

[24] Melchior F, Schergaut M, Pichler A (2003). SUMO: ligases, isopeptidases and nuclear pores. Trends Biochem Sci.

[25] Geiss-Friedlander R, Melchior F (2007). Concepts in sumoylation: a decade on. Nat Rev Mol Cell Biol.

[26] Johnson ES (2004). Protein modification by SUMO. Annu Rev Biochem.

[27] Mukhopadhyay D, Dasso M (2007). Modification in reverse: the SUMO proteases. Trends Biochem Sci.

[28] Hay RT (2007). SUMO-specific proteases: a twist in the tail. Trends Cell Biol.

[29] Reverter D, Lima CD (2004). A basis for SUMO protease specificity provided by analysis of human Senp2 and a Senp2-SUMO complex. Structure.

[30] Shen LN, Dong C, Liu H, Naismith JH, Hay RT (2006). The structure of SENP1-SUMO-2 complex suggests a structural basis for discrimination between SUMO paralogues during processing. Biochem J.

[31] Hang J, Dasso M (2002). Association of the human SUMO-1 protease SENP2 with the nuclear pore. J Biol Chem.

[32] Zhang H, Saitoh H, Matunis MJ (2002). Enzymes of the SUMO modification pathway localize to filaments of the nuclear pore complex. Mol Cell Biol.

[33] Goeres J, Chan PK, Mukhopadhyay D, Zhang H, Raught B, Matunis MJ (2011). The SUMO-specific isopeptidase SENP2 associates dynamically with nuclear pore complexes through interactions with karyopherins and the Nup107-160 nucleoporin subcomplex. Mol Biol Cell.

[34] Eifler K, Vertegaal AC (2015). SUMOylation-mediated regulation of cell cycle progression and cancer. Trends Biochem Sci.

[35] Bell SP, Dutta A (2002). DNA replication in eukaryotic cells. Annu Rev Biochem.

[36] Scholefield G, Veening JW, Murray H (2011). DnaA and ORC: more than DNA replication initiators. Trends Cell Biol.

[37] Prasanth SG, Prasanth KV, Siddiqui K, Spector DL, Stillman B (2004). Human Orc2 localizes to centrosomes, centromeres and heterochromatin during chromosome inheritance. EMBO J.

[38] Prasanth SG, Shen Z, Prasanth KV, Stillman B (2010). Human origin recognition complex is essential for HP1 binding to chromatin and heterochromatin organization. Proc Natl Acad Sci U S A.

[39] Prasanth SG, Prasanth KV, Stillman B (2002). Orc6 involved in DNA replication, chromosome segregation, and cytokinesis. Science.

[40] Lee KY, Bang SW, Yoon SW, Lee SH, Yoon JB, Hwang DS (2012). Phosphorylation of ORC2 protein dissociates origin recognition complex from chromatin and replication origins. J Biol Chem.

[41] Shen Z, Chakraborty A, Jain A, Giri S, Ha T, Prasanth KV, Prasanth SG (2012). Dynamic association of ORCA with prereplicative complex components regulates DNA replication initiation. Mol Cell Biol.

[42] Huang C, Cheng J, Bawa-Khalfe T, Yao X, Chin YE, Yeh ET (2016). SUMOylated ORC2 recruits a histone demethylase to regulate centromeric histone modification and genomic stability. Cell Rep.

[43] Azuma Y, Arnaoutov A, Anan T, Dasso M (2005). PIASy mediates SUMO-2 conjugation of Topoisomerase-II on mitotic chromosomes. EMBO J.

[44] Cubenas-Potts C, Goeres JD, Matunis MJ (2013). SENP1 and SENP2 affect spatial and temporal control of sumoylation in mitosis. Mol Biol Cell.

[45] Brito DA, Rieder CL (2006). Mitotic checkpoint slippage in humans occurs via cyclin B destruction in the presence of an active checkpoint. Curr Biol.

[46] Mihaylov IS, Kondo T, Jones L, Ryzhikov S, Tanaka J, Zheng J, Higa LA, Minamino N, Cooley L, Zhang H (2002). Control of DNA replication and chromosome ploidy by geminin and cyclin A.. Mol Cell Biol.

[47] Davoli T, Denchi EL, de Lange T (2010). Persistent telomere damage induces bypass of mitosis and tetraploidy. Cell.

[48] Shimada K, Pasero P, Gasser SM (2002). ORC and the intra-S-phase checkpoint: a threshold regulates Rad53p activation in S phase. Genes Dev.

[49] Deng Z, Dheekollu J, Broccoli D, Dutta A, Lieberman PM (2007). The origin recognition complex localizes to telomere repeats and prevents telomere-circle formation. Curr Biol.

[50] Tatsumi Y, Ezura K, Yoshida K, Yugawa T, Narisawa-Saito M, Kiyono T, Ohta S, Obuse C, Fujita M (2008). Involvement of human ORC and TRF2 in pre-replication complex assembly at telomeres. Genes Cells.

[51] Shimada K, Gasser SM (2007). The origin recognition complex functions in sister-chromatid cohesion in Saccharomyces cerevisiae. Cell.

[52] Pflumm MF, Botchan MR (2001). Orc mutants arrest in metaphase with abnormally condensed chromosomes. Development.

[53] Mukhopadhyay D, Dasso M (2010). The fate of metaphase kinetochores is weighed in the balance of SUMOylation during S phase. Cell Cycle.

[54] Watts FZ (2007). The role of SUMO in chromosome segregation. Chromosoma.

[55] Nie M, Xie Y, Loo JA, Courey AJ (2009). Genetic and proteomic evidence for roles of Drosophila SUMO in cell cycle control, Ras signaling, and early pattern formation. PLoS One.

[56] Zhang XD, Goeres J, Zhang H, Yen TJ, Porter AC, Matunis MJ (2008). SUMO-2/3 modification and binding regulate the association of CENP-E with kinetochores and progression through mitosis. Mol Cell.

[57] Agostinho M, Santos V, Ferreira F, Costa R, Cardoso J, Pinheiro I, Rino J, Jaffray E, Hay RT, Ferreira J (2008). Conjugation of human topoisomerase 2 alpha with small ubiquitin-like modifiers 2/3 in response to topoisomerase inhibitors: cell cycle stage and chromosome domain specificity. Cancer Res.

[58] Diaz-Martinez LA, Gimenez-Abian JF, Azuma Y, Guacci V, Gimenez-Martin G, Lanier LM, Clarke DJ (2006). PIASgamma is required for faithful chromosome segregation in human cells. PLoS One.

[59] Ryu H, Azuma Y (2010). Rod/Zw10 complex is required for PIASy-dependent centromeric SUMOylation. J Biol Chem.

[60] Dawlaty MM, Malureanu L, Jeganathan KB, Kao E, Sustmann C, Tahk S, Shuai K, Grosschedl R, van Deursen JM (2008). Resolution of sister centromeres requires RanBP2-mediated SUMOylation of topoisomerase IIalpha. Cell.

[61] Ryu H, Furuta M, Kirkpatrick D, Gygi SP, Azuma Y (2010). PIASy-dependent SUMOylation regulates DNA topoisomerase IIalpha activity. J Cell Biol.

[62] Klein UR, Haindl M, Nigg EA, Muller S (2009). RanBP2 and SENP3 function in a mitotic SUMO2/3 conjugation-deconjugation cycle on Borealin. Mol Biol Cell.

[63] Mukhopadhyay D, Arnaoutov A, Dasso M (2010). The SUMO protease SENP6 is essential for inner kinetochore assembly. J Cell Biol.

[64] Garcia-Rodriguez N, Wong RP, Ulrich HD (2016). Functions of ubiquitin and SUMO in DNA replication and replication stress. Front Genet.

[65] Mendez J, Stillman B (2000). Chromatin association of human origin recognition complex, cdc6, and minichromosome maintenance proteins during the cell cycle: assembly of prereplication complexes in late mitosis. Mol Cell Biol.

[66] Huang C, Han Y, Wang Y, Sun X, Yan S, Yeh ET, Chen Y, Cang H, Li H, Shi G, Cheng J, Tang X, Yi J (2009). SENP3 is responsible for HIF-1 transactivation under mild oxidative stress via p300 de-SUMOylation. EMBO J.

[67] Franken NA, Rodermond HM, Stap J, Haveman J, van Bree C (2006). Clonogenic assay of cells *in vitro*. Nat Protoc.

[68] Han Y, Huang C, Sun XX, Xiang BG, Wang M, Yeh ET, Chen YY, Li H, Shi GY, Cang H, Sun YP, Wang J, Wang W (2010). SENP3-mediated de-conjugation of SUMO2/3 from promyelocytic leukemia is correlated with accelerated cell proliferation under mild oxidative stress. J Biol Chem.

