# Supplementary material for: Reversible regulation of ORC2 SUMOylation by PIAS4 and SENP2
Source: Oncotarget. 2017 Jul 26;8(41):70142–55. doi: 10.18632/oncotarget.19594 (PMC5642542; doi:10.18632/oncotarget.19594)
Supplement: Supplementary file 1 [file oncotarget-08-70142-s001.pdf]

Reversible regulation of ORC2 SUMOylation by PIAS4 and SENP2

SUPPLEMENTARY MATERIALS

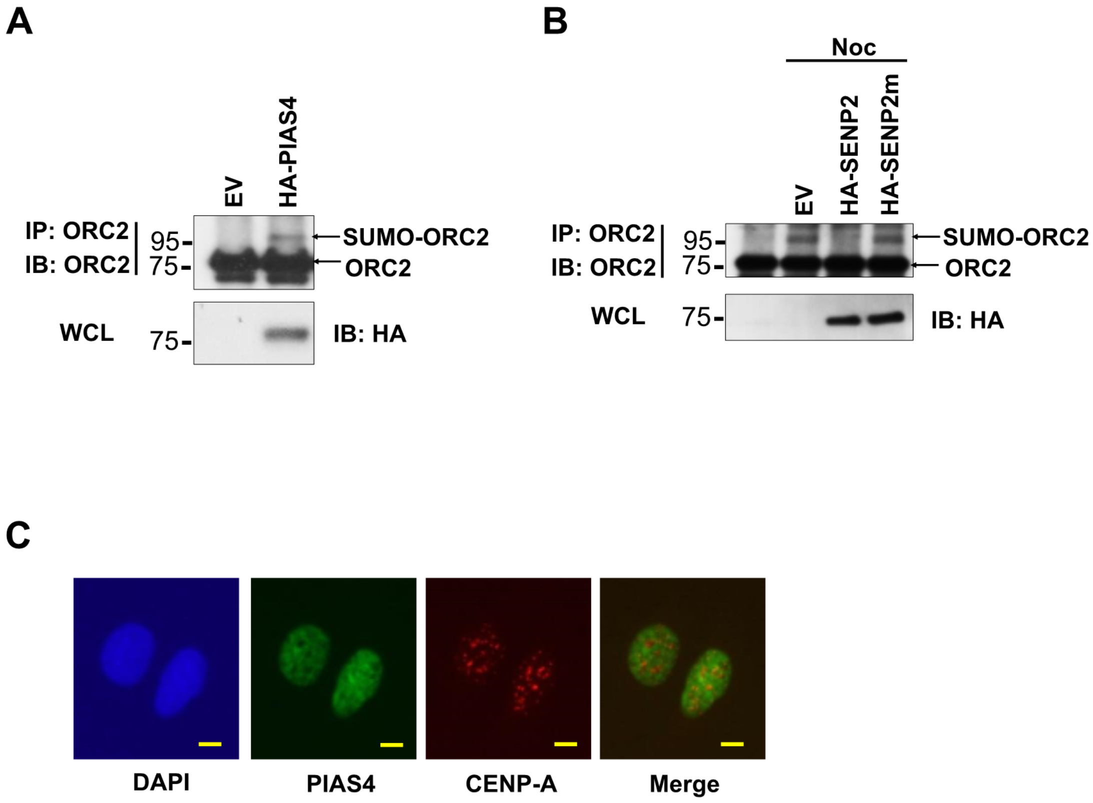

Supplementary Figure 1: Regulation of endogenous ORC2 SUMOylation by overexpressed SENP2 and PIAS4. (A) U2OS cells were transfected with HA-PIAS4 plasmids. Cells were treated with nocodazole for 24 hour. Endogenous ORC2 was pulled down by ORC2 antibody and blotted with ORC2 antibody. Whole cell lysates (WCL) were blotted with HA antibody. **(B)** U2OS cells were transfected with indicated plasmids. Endogenous ORC2 was pulled down by ORC2 antibody and blotted with ORC2 antibody. Whole cell lysates (WCL) were blotted with HA antibody. **(C)** U2OS cells were treated with nocodazole for 20 hours and then were released into fresh medium for 2 hours. Immunostaining was performed with PIAS4 antibody (green) and CENP-A antibody (red). Nuclei were stained with DAPI (blue). Scale bar: 10uM.

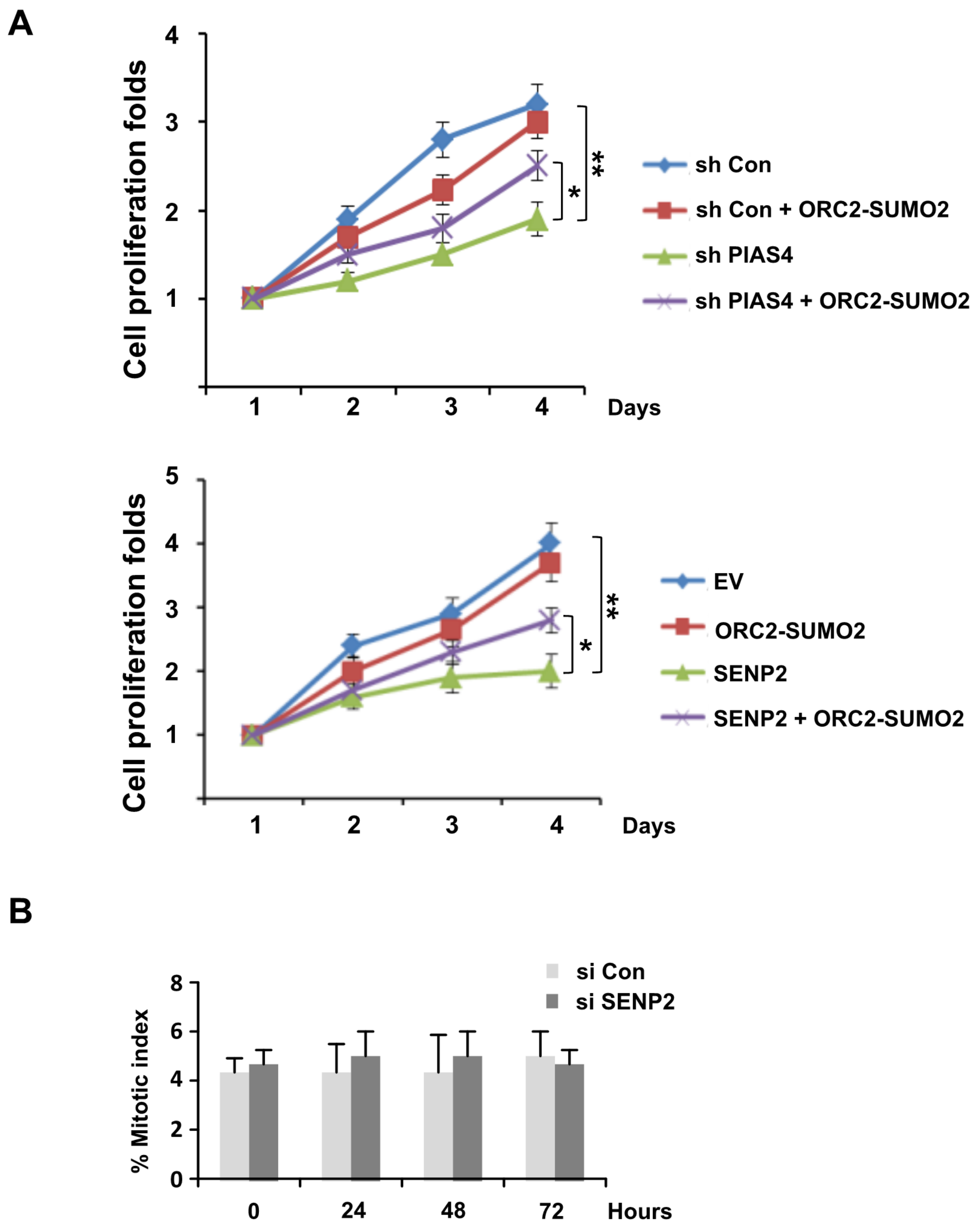

Supplementary Figure 2: ORC2 SUMOylation in control of cell cycle progression. **(A)** U2OS cells as in Figure 6B were subjected to MTT assay. The graph shows the average of three independent experiments; mean ± SD. *, $p < 0.05$; **, $p < 0.01$. **(B)** SENP2 in U2OS cells was knocked down as in Figure 1D. Mitotic index were determined by fluorescence microscopy analysis of DAPI-stained cells at different hours after transfection. The graph shows the average of three independent experiments; mean ± SD. $P>0.05$.
